# Two-dimensional monolayer salt nanostructures can spontaneously aggregate rather than dissolve in dilute aqueous solutions

**DOI:** 10.1038/s41467-021-25938-0

**Published:** 2021-09-23

**Authors:** Wenhui Zhao, Yunxiang Sun, Weiduo Zhu, Jian Jiang, Xiaorong Zhao, Dongdong Lin, Wenwu Xu, Xiangmei Duan, Joseph S. Francisco, Xiao Cheng Zeng

**Affiliations:** 1 Department of Physics, Ningbo University, Ningbo, 315211 China; 2 Department of Materials Science and Engineering, University of Science and Technology of China, Hefei, Anhui 230026 China; 3 Department of Chemistry, University of Nebraska-Lincoln, Lincoln, NE 68588 USA; 4 Department of Earth and Environmental Science, University of Pennsylvania, Philadelphia, PA 19104 USA; 5 Department of Chemistry, University of Pennsylvania, Philadelphia, PA 19104 USA; 6 Department of Chemical & Biomolecular Engineering, University of Nebraska-Lincoln, Lincoln, NE 68588 USA

**Keywords:** Physical chemistry, Chemical physics, Computational chemistry, Molecular dynamics

## Abstract

It is well known that NaCl salt crystals can easily dissolve in dilute aqueous solutions at room temperature. Herein, we reported the first computational evidence of a novel salt nucleation behavior at room temperature, i.e., the spontaneous formation of two-dimensional (2D) alkali chloride crystalline/non-crystalline nanostructures in dilute aqueous solution under nanoscale confinement. Microsecond-scale classical molecular dynamics (MD) simulations showed that NaCl or LiCl, initially fully dissolved in confined water, can spontaneously nucleate into 2D monolayer nanostructures with either ordered or disordered morphologies. Notably, the NaCl nanostructures exhibited a 2D crystalline square-unit pattern, whereas the LiCl nanostructures adopted non-crystalline 2D hexagonal ring and/or zigzag chain patterns. These structural patterns appeared to be quite generic, regardless of the water and ion models used in the MD simulations. The generic patterns formed by 2D monolayer NaCl and LiCl nanostructures were also confirmed by ab initio MD simulations. The formation of 2D salt structures in dilute aqueous solution at room temperature is counterintuitive. Free energy calculations indicated that the unexpected spontaneous salt nucleation behavior can be attributed to the nanoscale confinement and strongly compressed hydration shells of ions.

## Abstract (web summary)

Aqueous solutions under nanoscale confinement exhibit interesting physicochemical properties. This work reports evidence on the spontaneous formation of two-dimensional alkali chloride crystalline/non-crystalline nanostructures in dilute aqueous solution under nanoscale confinement by computer simulations.

## Introduction

Aqueous solutions under nanoscale confinement have attracted considerable interest over the past few years, owing to their unusual structural, dynamical, and physicochemical properties (different from those of their bulk counterparts), as well as to their broad significance for nanoscale chemical, biological, and physical systems such as ion channels/batteries and water desalination^1–15^. For instance, numerous experiments and molecular dynamics (MD) simulations revealed that nanoconfined water may freeze into various one-dimensional (1D) and two-dimensional (2D) polymorphous and polyamorphous structures at low temperatures^4,8,10,16–29^. Fast mass transport and high proton conductivity were also observed for water confined inside nanotubes^2,7,30^. Notably, previous computational and experimental studies demonstrated that the static dielectric constant of water can exhibit marked decrease under strongly confined conditions^31–33^, which may induce unusual behaviors of ions in the strongly confined water. Jiang and co-workers reported that when the Na^+^ ion was hydrated by three water molecules, its diffusion on the NaCl (001) surface was orders of magnitude faster than that of other hydrated ions^34^. Very recently, based on in situ graphene liquid cell transmission electron microscopy measurements, Wang et al. found that when a saturated NaCl solution was confined into graphene nanocapillaries, it formed rock salt NaCl with an intriguing hexagonal morphology^35^. In addition, a metastable hexagonal NaCl phase was detected during the crystallization process. These novel crystallization behaviors reflected a delicate interplay between graphene–solute interactions and thermodynamic behavior under nanoscale confinement.

It is well known that alkali chloride salts can dissolve spontaneously in bulk water and form hydrated ions^36,37^. Previous MD simulations provided molecular-level insights into the dissolution dynamics of NaCl nanocrystals in water^38–41^. Wang and co-workers found that upon placing a NaCl nanocrystal in bulk water, the water coordination number of the surface ions fluctuated with considerable amplitude during the hydration process, and the hydration interaction was the microscopic driving force for the dissolution of the NaCl nanocrystal in water^39^. The authors found that Cl^−^ ions at the corner of the NaCl nanocrystal tended to dissolve into water first, followed by an adjacent Na^+^ ion^39,40^. This sequence of ions with alternating charge dominated the dissolution process of the NaCl nanocrystal in bulk water. Their results also showed that ion dissolution was accompanied by dynamical transformations of the hydration shells and instantaneous fluctuations of the local water density^39^. Ab initio molecular dynamics (AIMD) simulations also showed that the dissolution of NaCl nanocrystals started at corner sites^41^.

For aqueous ionic solutions confined into nanopores, previous computational and experimental studies demonstrated that the nanoscale confinement can result in the ion hydration shells to be partially broken (i.e., leading to ion dehydration)^5,34,42–45^. The restricted hydrated structure of Rb and Br ions confined in carbon nanospaces was detected by using the extended X-ray absorption fine structure (EXAFS)^46^. The dehydration of ions within the nanopores may lead to unusual physical behaviors, not observed in the corresponding bulk solutions^34,42–50^. Either suppressed or enhanced ionic mobilities were reported for aqueous ionic solutions confined into nanopores of different size^7,45,50,51^. MD simulations showed that Li^+^ and Na^+^ ions can diffuse faster than water molecules in nanoslits, owing to frequent lateral hopping of the ions inside the bilayer solid-like water phase^44^. Local ion accumulation under inhomogeneous nanoconfinement was observed at the boundaries between coexisting water phases^48^. On graphene surfaces, strong hydrated cation–*π* interactions can induce the spontaneous formation of 2D NaCl crystals with unconventional non-1:1 stoichiometries (i.e., Na_2_Cl and Na_3_Cl) from dilute solutions at room temperature^13^. In addition, exotic hexagonal NaCl thin films were observed on the (110) diamond surface in absence of water^52^.

In this work, we reported the spontaneous nucleation of monolayer NaCl and LiCl nanostructures within water confined between two smooth hydrophobic walls at room temperature. Interestingly, monolayer NaCl nanocrystals adopted a square-unit pattern, while monolayer LiCl nanostructures exhibited non-crystalline hexagonal ring and/or zigzag chain patterns. The stability of the monolayer NaCl and LiCl nanostructures was confirmed by classical MD simulations with several different force fields, as well as AIMD simulations. To the best of our knowledge, this was the first computational evidence of nanoscale confinement inducing spontaneous nucleation of monolayer salt nanostructures in dilute solutions at room temperature.

## Results

### The spontaneous nucleation of monolayer NaCl and LiCl nanostructures within confined water

**Fig. 1:**
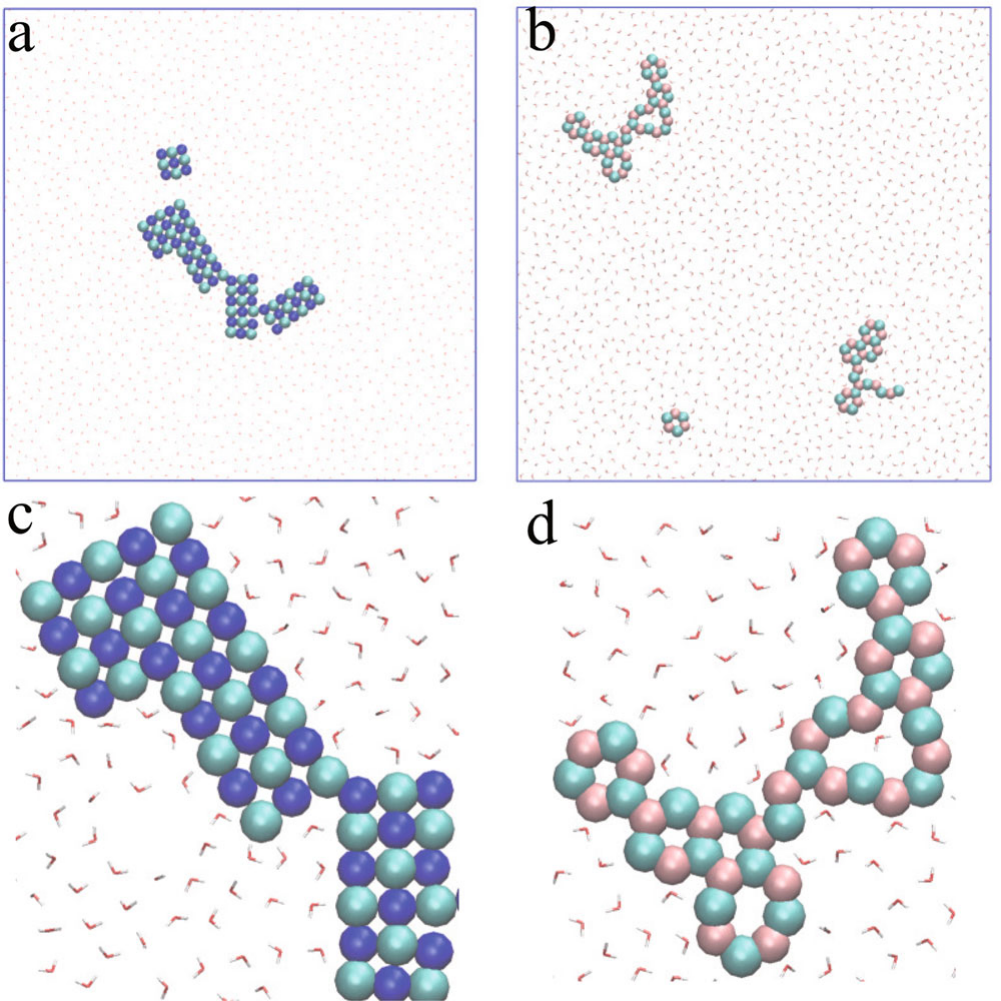
Monolayer salt nanostructures. **a**, **b** Top views of snapshots of **a** monolayer NaCl nanocrystals (polycrystals) and **b** LiCl non-crystalline nanostructures formed within confined water at the end of 1 μs simulations using the TIP4P^53^ and Charmm27 force fields for water and ions, respectively. **c**, **d** Enlarged views of **c** polycrystalline domains of 2D NaCl nanocrystal and **d** non-crystalline nanostructure of 2D LiCl monolayer. Water molecules were displayed as red-white lines, whereas Na^+^, Li^+^, and Cl^−^ ions were represented as blue, pink, and cyan spheres, respectively.

First, microsecond-scale MD simulations of dilute alkali chloride (i.e., NaCl or LiCl) aqueous solutions confined between two smooth hydrophobic walls were performed in the *NP*_*xy*_*T* ensemble. The system contained 1520 water molecules, 40 anions (Cl^−^), and 40 cations (Na^+^ or Li^+^), corresponding to a concentration (molality, *m*) of 1.46 mol/kg. The latter (expressed in mol_ion_/kg_H2O_) was much lower than the experimental solubility of sodium chloride (6.15 mol/kg) at room temperature. Initially, the ions randomly dissolved in the confined water at room temperature (as shown in Supplementary Fig. 1). After equilibration for 1 μs, spontaneous formation of phase-separated NaCl (or LiCl) domains was observed within the confined water, as shown in Fig. 1 and Supplementary Movies 1 and 2. Remarkably, Fig. 1a showed that Na^+^ and Cl^−^ ions aggregated to form monolayer nanocrystals with square units, identical to the typical monolayer structure of bulk B1-NaCl crystals. In stark contrast to the square-unit pattern of the NaCl nanocrystals, the LiCl domains were composed of hexagonal rings and/or zigzag chains (as shown in Fig. 1b), suggesting non-crystalline rather than crystalline nanostructures. Interestingly, the LiCl domain with hexagonal pattern was similar to the hexagonal NaCl monolayer formed on the (110) diamond surface^52^. Moreover, no isolated ions were observed in the aqueous solution in the final configuration of the simulation; in other words, the ions were fully dehydrated. The transverse density profiles (TDPs, Supplementary Fig. 2) showed that the ionic nanostructures within the confined water exhibited a unimodal distribution, while the water layer showed a bimodal distribution indicating a puckered structure.

**Fig. 2:**
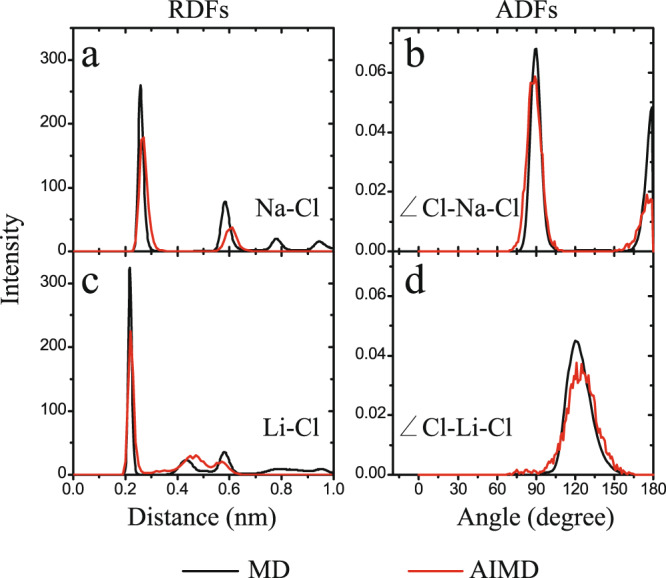
Structural features of monolayer salt nanostructures. Radial distribution functions (RDFs) (**a**, **c**) and angle distribution function (ADFs) (**b**, **d**) for NaCl (top) and LiCl (bottom) monolayer nanostructures. The black and red curves were obtained from classical molecular dynamics (MD) and ab initio molecular dynamics (AIMD) simulations, respectively.

To gain further insight into the structural features of the monolayer salt nanostructures, we calculated the lateral ion–ion radial distribution functions (RDFs) displayed in Fig. 2 and Supplementary Fig. 3. The calculated RDFs showed sharp peaks, indicating long-range order (Fig. 2a) and further confirming the nanocrystalline structure of the 2D NaCl monolayer. The Cl–Na–Cl angle distribution function (ADF, Fig. 2b) exhibited two peaks around 90° and 180°, corresponding to the square-unit pattern characterizing the 2D nanocrystalline structure of NaCl. The first peak of the Na–Cl RDF was located at ~0.26 nm, which was close to the Na–Cl bond length in the bulk B1-NaCl crystal (Fig. 2a), while the second peak was located at ~0.58 nm, corresponding to $\sqrt{5}\times 0.26$ nm. Moreover, Supplementary Fig. 3a showed that the first peaks of the Na–Na and Cl–Cl RDFs were both located at ~0.38 nm (=$\sqrt{2}\times 0.26$ nm). These results confirmed that the square-unit pattern dominated the underlying lattice structure of 2D NaCl nanocrystals.

The RDFs of the 2D monolayer LiCl nanostructures, in contrast, exhibited broad peaks (except for the first one), reflecting non-crystalline features (Fig. 2c). Moreover, the Cl–Li–Cl angle distribution function exhibited only one broad peak at ~120° (Fig. 2d), suggesting that the non-crystalline structure comprised numerous hexagonal rings. The first peak of the Li–Cl RDF was located at ~0.23 nm, close to the Li–Cl bond length in the bulk LiCl crystal (Fig. 2c), while the second peak was observed at ~0.45 nm, approximately equal to 2 × 0.23 nm. Furthermore, Supplementary Fig. 3b showed that the first peaks of both the Li–Li and Cl–Cl RDFs occurred at ~0.39 nm (=$\sqrt{3}\times 0.23$) nm, consistent with a Cl–Li–Cl angle of 120° (i.e., the non-crystalline monolayer LiCl nanostructure contained numerous hexagonal rings).

If the 2D LiCl domains exhibited a square-unit pattern similar to NaCl ones, the Cl–Cl distance would be expected to be equal to $\sqrt{2}\times 0.23$ nm (i.e., 0.32 nm), much smaller than 0.4 nm (≈L–J parameter *σ* of the Cl^−^ ion, as shown in Supplementary Table 1), indicating a strong repulsive interaction. This analysis may explain why the LiCl solid domains favored hexagonal rings rather than a square-unit arrangement.

Once all cations/anions in the dilution solution were used up, no further growth of the monolayer nanostructures was possible in the MD simulations. Nevertheless, the nanostructures still behaved like Brownian particles and self-diffused within the confined water, as indicated by the calculated lateral mean square displacements (MSDs), which increased linearly with time. The lateral diffusion coefficients of the ions were ~10^−5^ cm^2^/s; this value was lower than that of the water molecules, owing to the nucleation of ionic nanostructures (as shown in Supplementary Fig. 4).

Note that previous computational studies have shown that computed diffusion coefficients based on the mean-square displacement (MSD) can be dependent on the simulation-box size^54,55^. Here, we also analyzed the effect of the simulation-box size on the quantitative values of diffusion coefficient of ions and waters, and the structure of final assemblies. Consistent with previous studies^54,55^, the mean-square displacements exhibited system size dependence. However, the behavior of nucleation and growth of the monolayer ionic nanostructures was independent of the simulation-box size (Supplementary Fig. 4).

### The nucleation dynamics analysis of forming NaCl and LiCl monolayer nanostructures

**Fig. 3:**
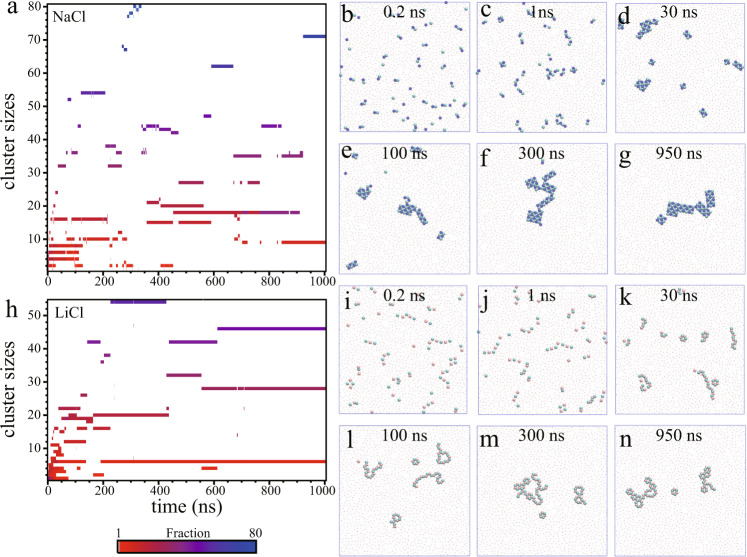
Nucleation and growth of monolayer salt nanostructures. Time-dependent distributions of ion cluster size (left panels, **a**, **h**) and representative snapshots (right panels, **b**–**g** and **i**–**n**) for 2D monolayer NaCl and LiCl nanostructures in dilute solutions. The definition of ion clusters is based on a distance criterion (i.e., an ion belongs to a cluster when its distance to any ion in the cluster is less than 0.35 nm).

To better understand the growth of the ionic monolayer nanostructures, the time evolutions of size distribution of the ion nuclei were displayed along with representative snapshots of ion domains extracted at various times from the MD simulations (as shown in Fig. 3a–g for NaCl and Fig. 3h–n for LiCl). We also investigated the number of cations with different Cl^−^ coordination number (*N*_*c*_) formed over the course of the simulation (Supplementary Fig. 5). As shown in Fig. 3a, most dissolved Na^+^ and Cl^−^ ions nucleated into ion dimers during the first 0.2 ns. In addition, small ion clusters (trimers, tetramers, pentamers, and hexamers) were observed within the confined water during the first 1 ns, as indicated by the rapid increase in the number of Na^+^ ions with *N*_*c*_ = 1 or 2 (Supplementary Fig. 5a). The ion tetramers and hexamers were arranged in a square-unit pattern comprising the same number of Na^+^ and Cl^−^ ions (Fig. 3c). The lifetimes of even-numbered ion clusters (dimers, tetramers, and hexamers) were of the order of tens of nanoseconds, while those of odd-numbered clusters (trimers and pentamers) were lower than 2 ns (Fig. 3a). Over the course of the simulation, the NaCl domains grew rapidly, as indicated by the rapid increase in the number of Na^+^ ions with *N*_*c*_ = 4 (Supplementary Fig. 5a). At ~310 ns, all 80 ions were aggregated into a sizable monolayer NaCl nanocrystal. This nanocrystal can be viewed as a polycrystal, composed of smaller crystallites with a square-unit pattern. Although the lifetime of the largest NaCl nanocrystal was only several nanoseconds, the smaller nanocrystals remained thermodynamically stable over tens to hundreds of nanoseconds. Interestingly, at the end of simulation we also observed a NaCl nonamer including five Na^+^ and four Cl^−^ ions. Although the nonamer domain was not electrically neutral, its lifetime was longer than 150 ns, owing to its unique 3 × 3 structure arranged in a square pattern (see the 950 ns snapshot in Fig. 3g and Supplementary Movie 1). Overall, the crystalline domains of monolayer NaCl nanocrystals appeared to be thermodynamically stable within the confined water.

For the LiCl solution, Fig. 3h showed that small-sized ion clusters (i.e., dimer, trimer, tetramer, pentamer, etc.) were formed in the first several nanoseconds of the MD simulation, as indicated by the rapid increase in the number of Li^+^ ions with *N*_*c*_ > 0 (Supplementary Fig. 5b). Unlike the NaCl solution, however, some isolated ions remained intact within the confined water during the first 75 ns (i.e., *N*_*c*_ = 0). These small-sized ion clusters only persisted for several nanoseconds, after which they aggregated into larger nanostructures composed of zigzag chains, hexagonal rings, or both (Fig. 3i–n). In particular, two types of structures were observed for the hexamers, i.e., a hexagonal ring and a zigzag chain composed of three Li^+^ and three Cl^−^ ions (Fig. 3k). The zigzag chain hexamer remained intact for the first several tens of nanoseconds, whereas its hexagonal ring counterpart persisted for over hundreds of nanoseconds, indicating the higher dynamic stability of this structure in the classical MD simulation. The largest LiCl domain (including 27 Li^+^ ions and 27 Cl^−^ ions, and composed of both zigzag chains and hexagonal rings) was observed at ~210 ns. This large non-crystalline LiCl nanostructure remained stable for 200 ns. During the final several hundred nanoseconds, a hexagonal ring and two larger non-crystalline nanostructures formed within the confined water. Supplementary Fig. 5b showed that the number of Li^+^ ions with *N*_*c*_ = 2 slowly decreased while that with *N*_*c*_ = 3 increased after 100 ns, indicating the growth of assembled hexagonal rings. Whether the formation of monolayer LiCl nanocrystal with hexagonal structure from the mixture of zigzag chains and hexagonal rings would occur or not require much longer simulation beyond 10^3^ ns, and this will be a subject of future study.

A key question was why all the largest nanostructures observed in the MD simulations had a size no larger than 80 ions and a very short lifetime (only a few nanoseconds). To address this question, we carried out two independent larger-scale MD simulations with 250 ion pairs (i.e., 250 anions and 250 cations) and 9500 water molecules (i.e., corresponding to the same molality of 1.46 mol_ion_/kg_H2O_). As shown in Supplementary Fig. 6, the largest NaCl nanocrystal formed during a MD simulation of 1 μs contained 292 ions arranged in a square-unit pattern. We also observed that the largest LiCl nanostructures included 104 ions, arranged in both hexagonal ring and zigzag chain patterns (Supplementary Fig. 7). The two larger-scale simulations thus showed that larger monolayer ionic nanostructures with longer lifetimes can form in larger systems.

To confirm that the spontaneous formation of ionic nanostructures within water confined between two walls was independent of the molecular model selected for the simulations, we performed a series of nine independent MD simulations using Charmm27, OPLS, and Amber03 force fields for the ions, along with TIP4P^53^, TIP3P^56^, and SPC/E^57^ models for water. These simulations were performed both as benchmark tests and to compare their results with those obtained using the TIP4P and Charmm27 force fields for water and ions, respectively. Furthermore, we performed two additional independent MD simulations of ion solutions confined between two atomic-scale graphene sheets (in the *NVT* ensemble). As shown in Supplementary Figs. 8–10, all benchmark test simulations showed a qualitatively similar behavior; in other words, the spontaneous formation of ionic monolayers appeared to be a general phenomenon occurring in highly confined dilute solutions, regardless of the molecular models selected.

### Estimation the stability of the ionic nanostructures using AIMD simulation

**Fig. 4:**
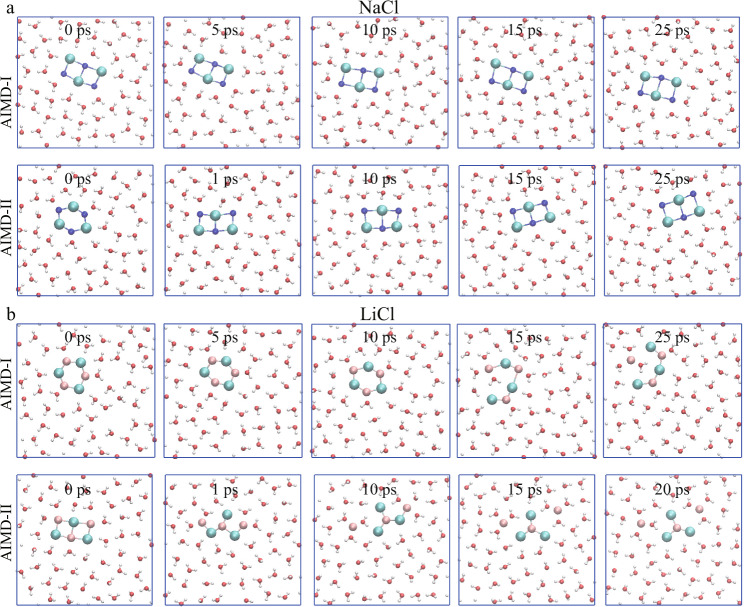
Ab initio molecular dynamics (AIMD) simulations. Snapshots of NaCl nanocrystal (**a**) and LiCl structures (**b**), confined between two graphene sheets. AIMD-I was started with the relatively stable initial configurations and AIMD-II was started with the unstable initial configurations. Water molecules were displayed as red–white lines, while Na^+^, Li^+^, and Cl^−^ were represented as blue, pink, and cyan spheres, respectively. The graphene walls were not shown.

To further confirm that the stability of the ionic nanostructures, AIMD simulations were carried out using the Quickstep module implemented in the CP2K package^58^. The initial configurations selected for these simulations were either a square-unit nanocrystal or a hexagonal ring placed within water confined between two graphene sheets (Fig. 4). For the NaCl system, the square-unit nanocrystal was intact without structural changes during the 25 ps AIMD simulation (see AIMD-I in Fig. 4a and Supplementary Movie 3). Moreover, another independent AIMD simulation starting from NaCl hexagonal ring was performed, where the initial hexagonal ring was converted into the apparently more stable square-unit structure within 1 ps (see AIMD-II in Fig. 4a and Supplementary Movie 4). These results indicated that the NaCl square-unit nanocrystal is likely the most stable structure within the confined water, consistent with the classical MD simulation results. For the LiCl system, the hexagonal ring structure remained stable more than 10 ps, followed by a structural conversion to the zigzag-like chain structure. During the remaining 10+ ps AIMD simulation, the zigzag-like chain was well kept (see AIMD-I in Fig. 4b and Supplementary Movie 5). Additionally, the LiCl square-unit nanocrystal (as the initial structure) transformed into a zigzag-like chain structure within 1 ps as well (see AIMD-II in Fig. 4b and Supplementary Movie 6). Due to the small system size used in the AIMD simulation (with only 3 ion pairs), the zigzag-like chain structure turned into a small-sized cluster with can be still viewed as a pre-critical cluster for the zigzag-chain structure. These results suggested that the single LiCl hexagonal ring is likely a metastable structure whereas the  zigzag-like chain structure is a relatively more stable structure. We also calculated the RDFs and ADFs based on the AIMD simulations. As shown in Fig. 2, the results were consistent with those obtained from classical MD, further confirming the stability of the ionic nanostructures within the confined water.

### The nucleation mechanism through free energy computation and ion hydrate analysis

**Fig. 5:**
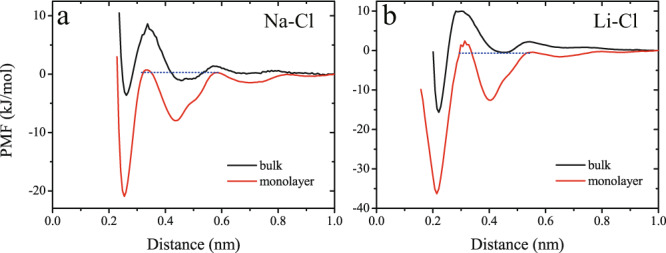
Free energy profiles. Potential of mean force (PMF) curves for **a** Na^+^/Cl^−^ and **b** Li^+^/Cl^−^ ion pairs in bulk and confined water.

To elucidate the nucleation mechanism of the ions within confined water, we calculated the potential of mean force (PMF) of ion pairs in both bulk and confined water. As shown in Fig. 5, the lowest free energy minima were located at Na–Cl and Li–Cl distances of 0.26 and 0.23 nm, consistent with the nearest-neighbor Na–Cl and Li–Cl distances in monolayer nanostructures, respectively (*i.e*., the first peaks in Fig. 2a, c). The aggregation kinetics of Na^+^ and Cl^−^, or Li^+^ and Cl^−^ ions within the confined water were significantly different from those in bulk water. The PMF curves showed that the free energy barriers for Na^+^ and Cl^−^ or Li^+^ and Cl^−^ ions approaching each other (from an initial distance larger than 0.80 nm to the first local free energy minimum at an interionic distance of ~0.42 nm) were similar to those observed for ions in bulk water. However, the local free energy minimum was much lower in the case of confined water than bulk water, indicating that the Na^+^/Cl^−^ or Li^+^/Cl^−^ ion pairs had a stronger tendency to aggregate within the confined water. Moreover, because the local free-energy barrier to ion pair dissociation was very low in the case of bulk water, the two opposite-charge ions had a much higher tendency to separate from one another than to form an ion dimer; conversely, the free energy barrier to ion pair dissociation was much higher in the case of confined water.

For confined water, the corresponding free energy barriers to ion pair association and dissociation starting from the local free energy minimum (at a distance of ~0.42 nm) were similar to each other. The global free energy minima (at ~0.26 nm for NaCl and 0.23 nm for LiCl) corresponded to the size of critical nuclei for the nucleation. The deeper free energy global minimum in the case of confined water indicated that the ion dimer state was more favorable. In other words, both the Na^+^/Cl^−^ and Li^+^/Cl^−^ ion pairs had a higher tendency to spontaneously form an ion dimer within confined water than bulk water. Finally, the free energy barrier to reach the global free energy minimum was lower for the Na^+^/Cl^−^ than the Li^+^/Cl^−^ pair. This explained why Na^+^ and Cl^−^ aggregated faster than Li^+^ and Cl^−^, and a higher number of fully hydrated Li^+^ ions persisted within the confined water during the first 75 ns, while most Na^+^ and Cl^−^ ions formed ion dimers within 0.2 ns (Fig. 3).

**Fig. 6:**
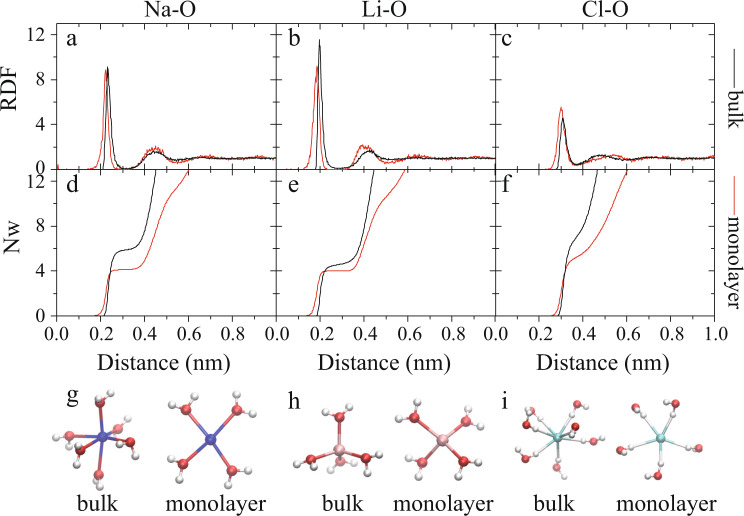
The ion hydrates in bulk and confined water. **a**–**c** Calculated ion–oxygen radial distribution functions (RDFs) and **d**–**f** water coordination numbers (*N*_*w*_) *vs*. interionic distance in bulk (black curve) and confined water (red curve) water. **g**–**i** Corresponding hydration shell structures of Na^+^, Li^+^, and Cl^−^ in bulk and confined water.

To further understand the different ion dimer formation tendencies of bulk and confined water, we investigated the corresponding solvation structures by calculating the ion–water RDFs and the water coordination numbers of the ions (*N*_*w*_). For this purpose, the interionic distance of the ion pairs was constrained at 1 nm to avoid their aggregation. As shown in Fig. 6, both coordination numbers in confined water (*N*_*w*_ = 4 for Na^+^ and 5 for Cl^−^) were smaller than those in bulk water (*N*_*w*_ = 6 for Na^+^ and 8 for Cl^−^). In addition, the *N*_*w*_ value of Li^+^ in confined water was 4. These reduced coordination numbers were attributed to the nanoscale confinement, under which the ion hydration shells became flattened. In other words, the nanoscale confinement effectively weakened the ionic hydration interaction, thereby inducing the spontaneous formation of ion nanostructures.

## Discussion

In conclusion, microsecond-scale MD simulations showed the spontaneous nucleation of 2D monolayer NaCl and LiCl nanostructures in highly confined dilute aqueous solutions at room temperature. This counterintuitive behavior was in stark contrast to that of bulk dilute solutions, in which salt NaCl crystals can easily dissolve in water at room temperature. The simulation results also showed that, in the confined water, NaCl tended to form monolayer nanocrystals with a square-unit pattern, while LiCl formed non-crystalline nanostructures composed of randomly distributed hexagonal rings and/or zigzag chains. The spontaneous formation of NaCl and LiCl nanostructures in confined water appeared to be a generic phenomenon, irrespective of the specific molecular models employed to represent water and ions. Three different force fields for ions (Charmm27, Amber03, and OPLSAA) showed qualitatively the same phenomenon, i.e., the spontaneous formation of monolayer NaCl and LiCl nanostructures in 2D water. AIMD simulations further confirmed the stability of the monolayer salt nanostructures in the confined water. The calculated potentials of mean force for cation/anion pairs showed that both the Na^+^/Cl^−^ and Li^+^/Cl^−^ pairs had a higher tendency to spontaneously form ion dimers within confined water than bulk water. This was because of the lower free energy barrier to reach the global free-energy minimum (PMF basin) and the deeper global free-energy minimum for the ion dimer state in the case of confined water. We envisaged that the spontaneous formation of 2D monolayer NaCl nanocrystals predicted in this study will simulate future experiments, e.g., focused on dilute NaCl solutions confined between graphene nanocapillaries^35^, to validate the present theoretical findings.

## Methods

### Classical molecular dynamics simulations

The classical MD simulations were carried out using the GROMACS 4.5 package^59^. The modeled systems consisted of aqueous ion solutions confined between two smooth walls (nanoslits). The ions were modeled using the Charmm27, Amber03, and OPLSAA force fields, to ensure that the results of the simulations were independent of the model employed. Water molecules were represented by the TIP4P^53^, TIP3P^56^, and SPC/E^57^ models. The Lennard–Jones (L–J) and electrostatic parameters of the ion and water models were listed in Supplementary Table 1. The solution–wall interactions were described by the L–J 10–4 potential function, corresponding to the integral of the L–J 12–6 potential of the graphene walls. Thus, a nanoslit with a width of 0.8 nm could accommodate one layer of aqueous solution at ambient pressure. Rigid graphene walls were also used to describe the ion–wall and water–wall interactions. The L–J parameters for carbon atoms were *σ*_*c*_ = 0.34 nm and *ε*_*c*_ = 0.3598 kJ/mol. Similar results from the simulations of solution confined between two graphene walls showed that the spontaneous formation of NaCl or LiCl nanostructures within confined water was not affected by the solution–wall interactions (as shown in Supplementary Fig. 10).

All MD simulations were performed in the constant lateral pressure and temperature (*NP*_*xy*_*T*) ensemble, with periodic boundary conditions in the lateral directions (*x* and *y*). The temperature and pressure were controlled by the Nosé–Hoover thermostat^60,61^ and Parrinello–Rahman barostat^62^, respectively. A cutoff of 1 nm was used for the L–J interactions, and long-range electrostatic interactions were treated by the slab-adapted Ewald sum method^63^.

### Free energy calculations

Potential of mean force (PMF) profiles for different ion pairs were calculated using the umbrella sampling algorithm^64^. The force constant adopted for the harmonic bias potential was 5000 kJ/(mol·nm), due to the strong interactions between cation and anion. The harmonic force was used to constrain the ions at distances of 0.15–1.0 nm, in increments of 0.01 or 0.1 nm to enhance the resolution and smoothness of the PMF. Each distance interval was sampled for 10 ns, and the data obtained from the last 5 ns were analyzed using the weighted histogram analysis method (WHAM)^65^.

### Ab initio molecular dynamics simulations

AIMD simulations were performed using the Quickstep module implemented in the CP2K package^58^. Ion–valence electron interactions were represented by Goedecker–Teter–Hutter (GTH) pseudopotentials^66,67^. The GTH-valence double-zeta-polarized Gaussian basis combined with a plane-wave basis set (with an energy cutoff of 280 Ry) was selected for the AIMD simulations. The Gaussian and augmented plane wave (GAPW) scheme was applied to obtain well-converged forces for the Na^+^ ion^47,68^. The BLYP exchange–correlation functional was used together with the Grimme dispersion correction (D3)^69^. A time step of 0.5 fs was used to ensure sufficient energy conservation for highly confined water systems. The temperature was maintained at 300 K in constant-temperature and constant-volume (*NVT*) AIMD simulations.

## Supplementary information

Supplementary Information

Description of Additional Supplementary Files

Supplementary Movie 1

Supplementary Movie 2

Supplementary Movie 3

Supplementary Movie 4

Supplementary Movie 5

Supplementary Movie 6

## Data Availability

The data that support the findings of this study are available from the corresponding authors on reasonable request.
