# Supplementary material for: Two-dimensional monolayer salt nanostructures can spontaneously aggregate rather than dissolve in dilute aqueous solutions
Source: Nat Commun. 2021 Sep 23;12:5602. doi: 10.1038/s41467-021-25938-0 (PMC8460741; doi:10.1038/s41467-021-25938-0)
Supplement: Supplementary file 1 — Supplementary Information [file 41467_2021_25938_MOESM1_ESM.pdf]

Supplementary Information

Two-dimensional monolayer salt nanostructures can spontaneously aggregate rather than dissolve in dilute aqueous solutions

Wenhui Zhao,[1,7] Yunxiang Sun, [1,7] Weiduo Zhu,[2,7] Jian Jiang,[3] Xiaorong Zhao,[1] Dongdong Lin,[1] Wenwu Xu,[1] Xiangmei Duan,[1] Joseph S. Francisco[4,5,*] and Xiao Cheng Zeng[3,6*]

[1]*Department of Physics, Ningbo University, Ningbo 315211, China*

[2]*Department of Materials Science and Engineering, University of Science and Technology of China, Hefei, Anhui 230026, China*

[3]*Department of Chemistry, University of Nebraska-Lincoln, Lincoln, Nebraska 68588, USA*

[4]*Department of Earth and Environmental Science, University of Pennsylvania, Philadelphia, PA, 19104, USA*

[5]*Department of Chemistry, University of Pennsylvania, Philadelphia, PA, 19104, USA*

[6]*Department of Chemical & Biomolecular Engineering, University of Nebraska-Lincoln, Lincoln, Nebraska 68588, USA*

[7]These authors contributed equally: W. Zhao, Y. Sun and W. Zhu

*Authors to whom correspondence should be addressed: frjoseph@sas.upenn.edu; xzeng1@unl.edu

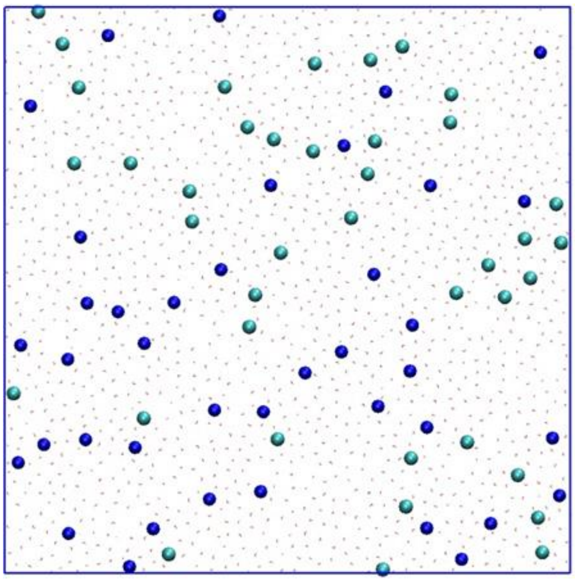

Supplementary Figure 1. A typical initial configuration with equal number of cations and anions randomly dissolved in monolayer water.

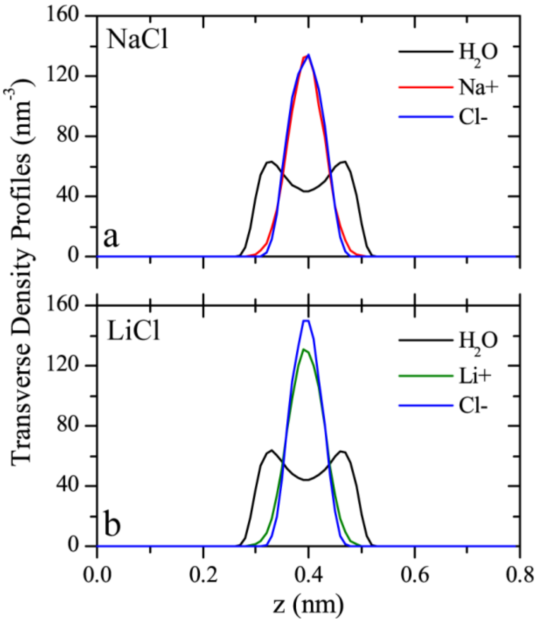

Supplementary Figure 2. Computed transverse density profiles (TDPs) of ions and water molecules confined to the nanoslit. To compare with water more clearly, the values for ions are multiplied by 38 (the ratio of the number of water molecules and ions).

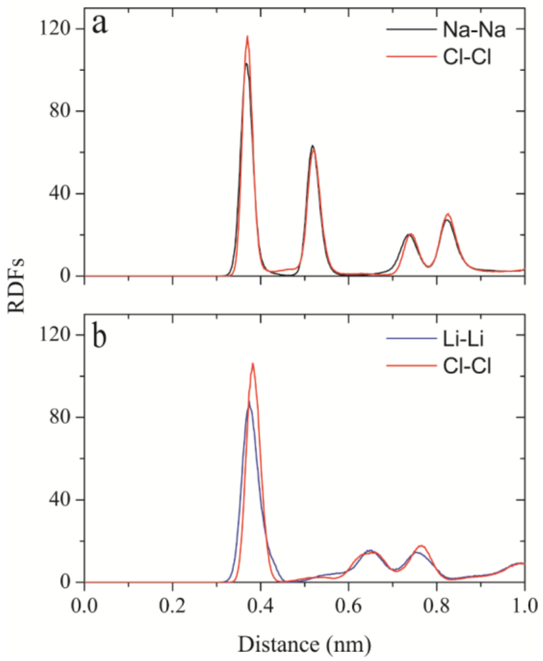

Supplementary Figure 3. Computed ion-ion RDFs for monolayer ion nanostructures in the monolayer water confined to the nanoslit.

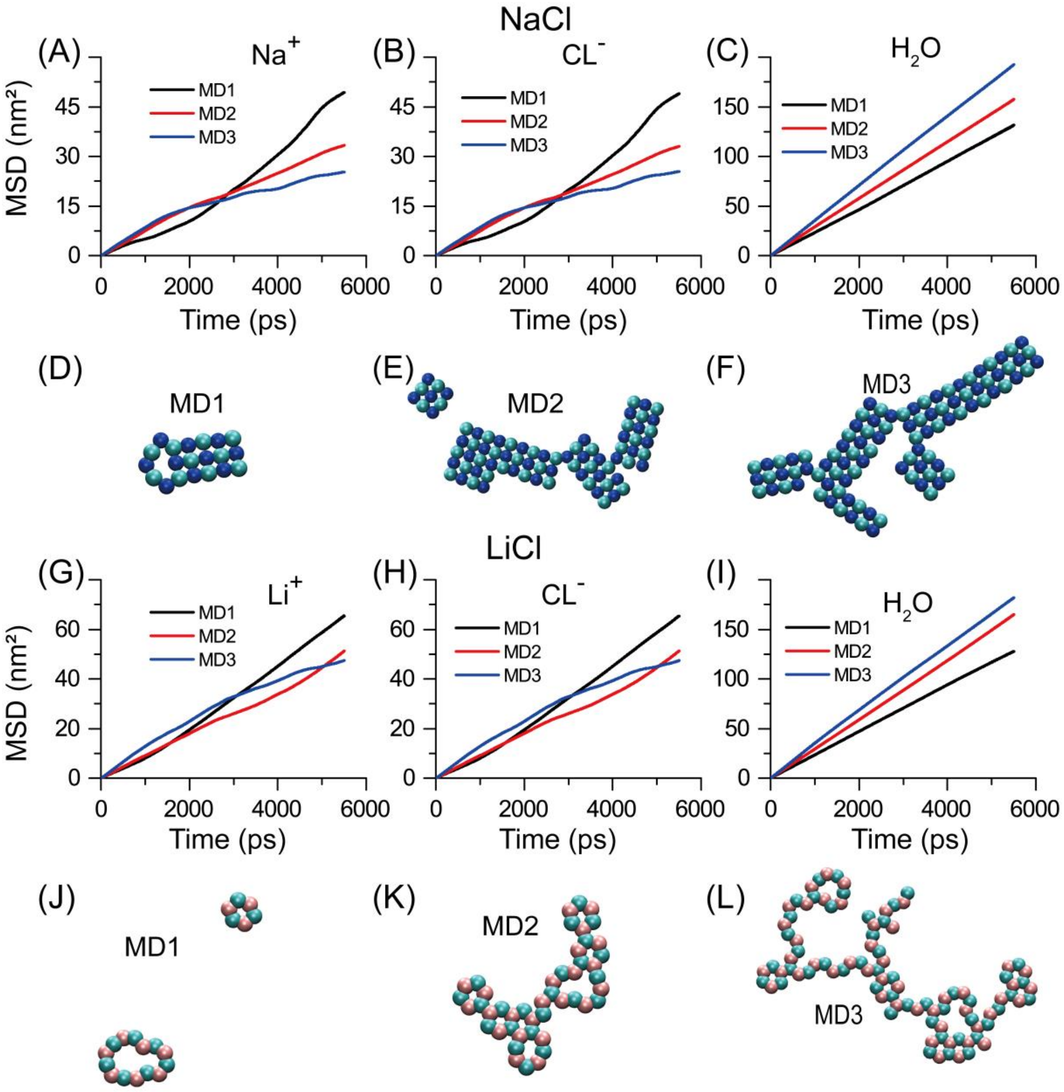

Supplementary Figure 4. Effect of different periodic boundary conditions on the computed diffusion coefficients of ions, waters, and final structure of assemblies, respectively. The lateral mean-square displacements (MSDs) of ions and water molecules obtained from three different MD simulations for Na^+-CL^- and Li^+-CL^- systems were shown in (A-C) and (G-I), respectively. The diffusion coefficients calculated based on the slope of MSD curves show system-size dependence. Final aggregates (for clarity, only one large cluster from final aggregates was present for each MD simulation) formed by same ions shared a similar conformation, indicating the conformation of Na^+-CL^- (D-F) and Li^+-CL^- (J-L) was independent of the simulation box size. The small-sized system of MD1 included 380 water molecules, 10 anions (Cl^-), and 10 cations (Na^+ or Li^+); the mid-sized system of MD2 included 1520 water molecules, 40 anions (Cl^-), and 40 cations (Na^+ or Li^+); while the largest system of MD3 included 9500 water molecules, 250 anions (Cl^-), and 250 cations (Na^+ or Li^+).

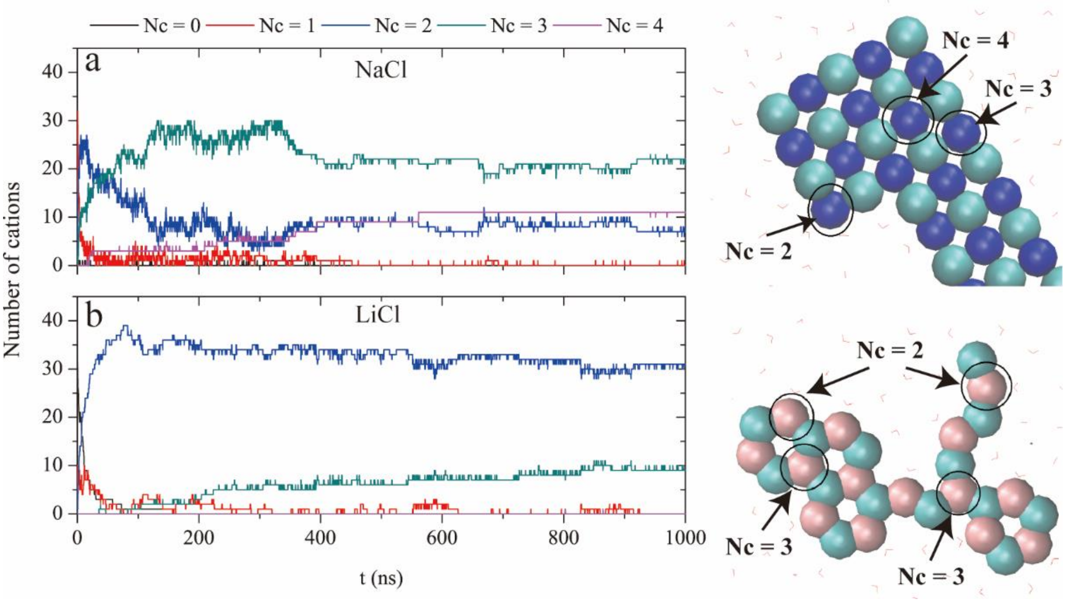

Supplementary Figure 5. Time dependent number of cations with different Cl^- coordination number (N_c) over the course of the MD simulations.

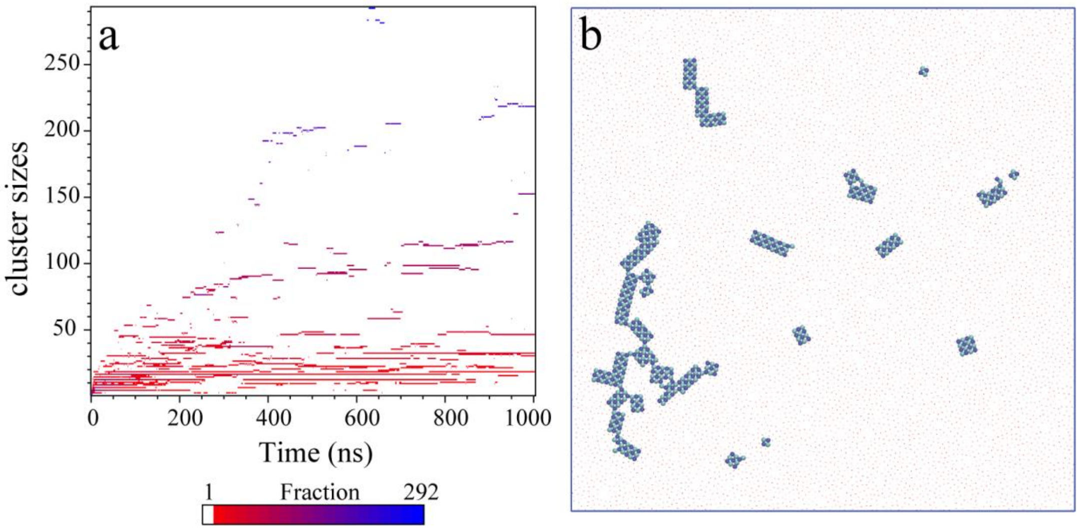

Supplementary Figure 6. (A) Time dependent distributions of cluster size of monolayer NaCl nanocrystal over the course of the simulations (the system had 250 ion pairs and 9500 water molecules confined between two smooth walls). (B) A snapshot of the monolayer NaCl nanocrystals with 292 ions at 628 ns.

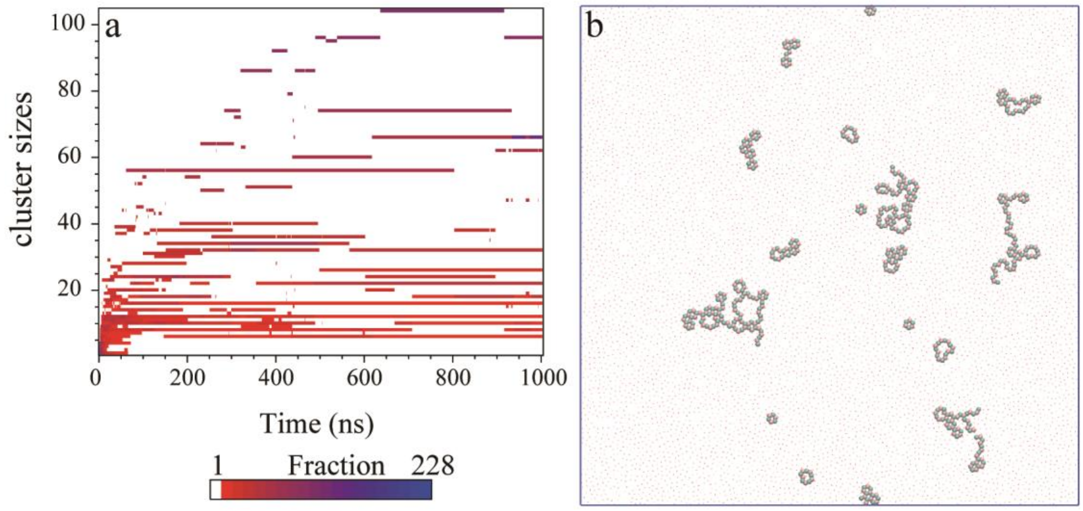

Supplementary Figure 7. (A) Time dependent distributions of cluster size of LiCl non-crystalline nanostructures over the course of the MD simulations (the system had 250 ion pairs and 9500 water molecules confined between two smooth walls). (B) A snapshot of LiCl nanostructures with 104 ions at 800 ns.

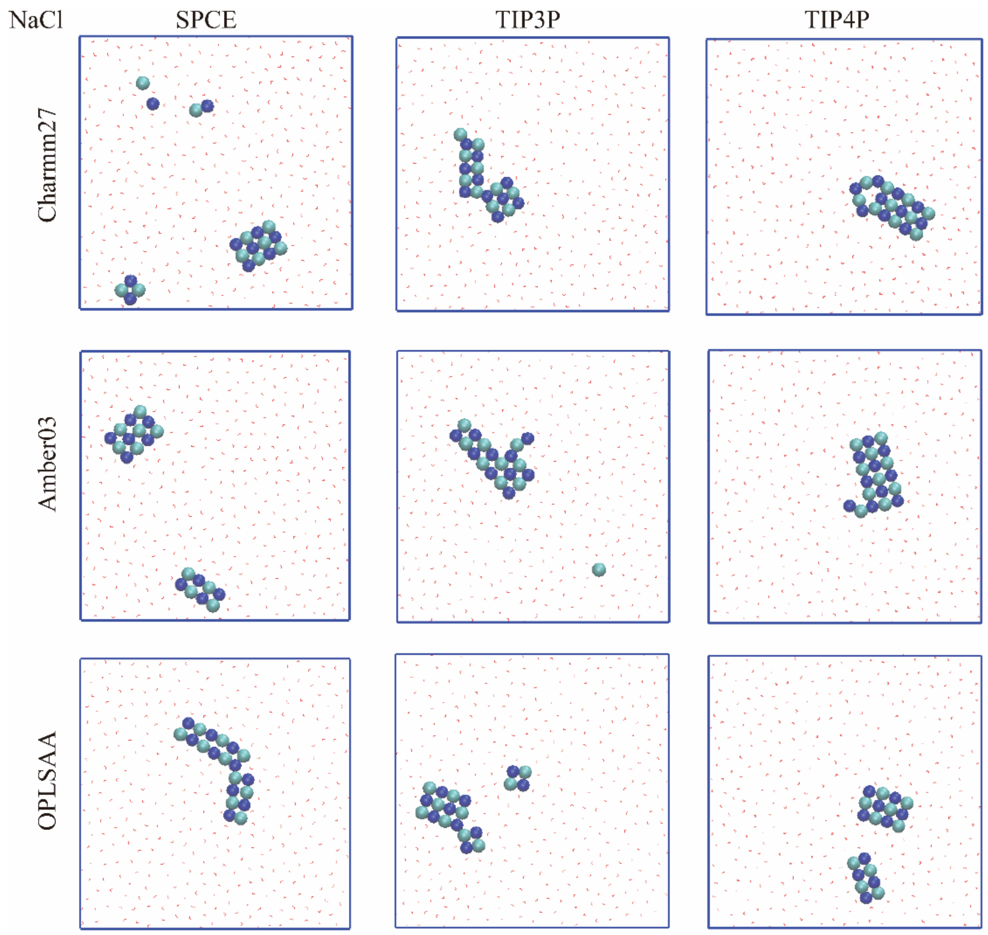

Supplementary Figure 8. Nine snapshots of monolayer NaCl nanocrystals in monolayer water, obtained by using Charmm27, Amber03 and OPLSAA force fields for ions and SPC/E, TIP3P and TIP4P water model, respectively, in nine independent MD simulations.

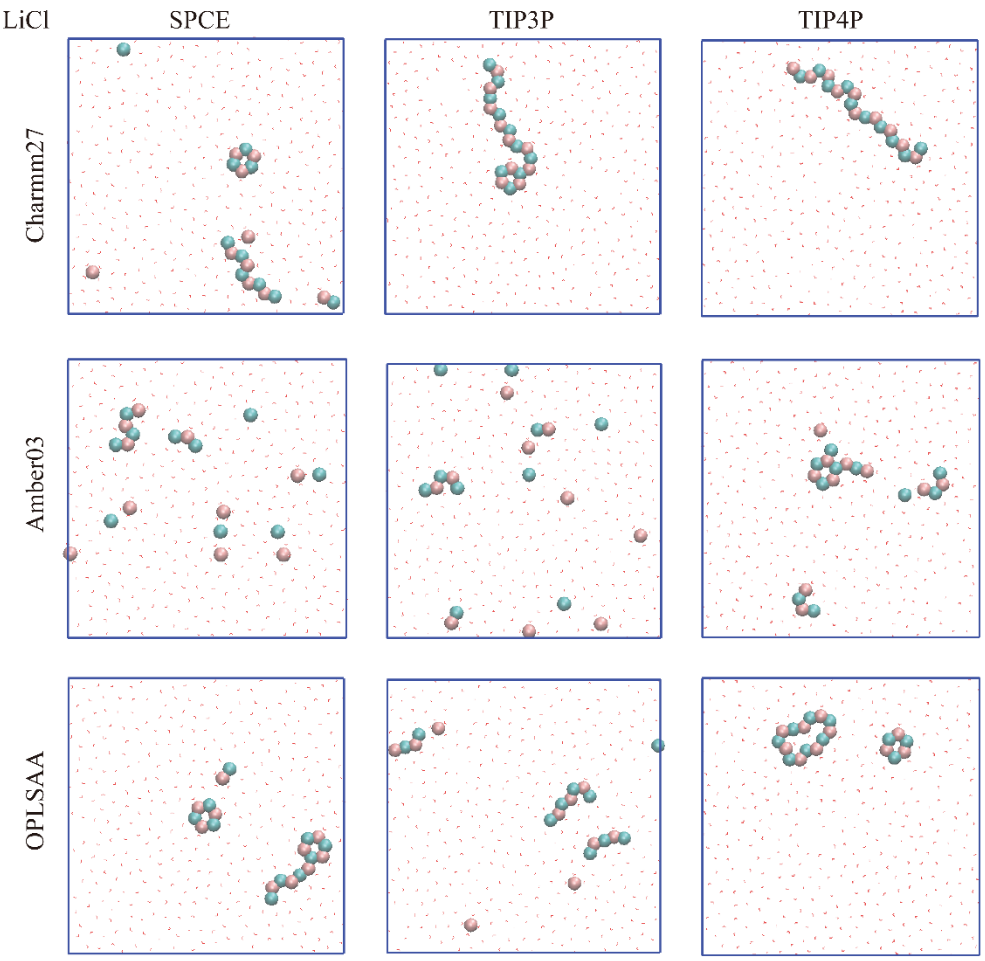

Supplementary Figure 9. Nine snapshots of LiCl monolayer nanostructures in monolayer water, obtained by using Charmm27, Amber03 and OPLSAA force fields for ions and SPC/E, TIP3P and TIP4P water model, respectively, in nine independent MD simulations.

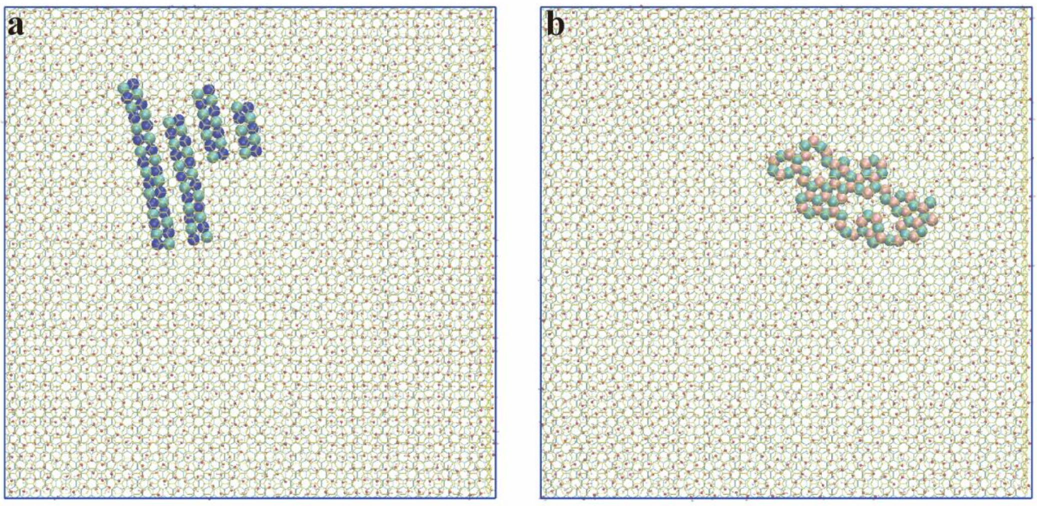

Supplementary Figure 10. Snapshots of (A) NaCl monolayer nanocrystal and (B) LiCl monolayer nanostructure confined between two atomistic graphene sheets with a relative rotation (Moire) angle of 30°. The carbon atoms were depicted as cyan (bottom layer) and yellow (upper layer) lines.

Supplementary Table 1. Lennard-Jones parameters for ions based on the Charmm27, Amber03 and OPLSAA force fields, respectively.

	Li^+		Na^+		Cl^-	
	σ (nm)	ε (kJ/mol)	σ (nm)	ε (kJ/mol)	σ (nm)	ε (kJ/mol)
Charmm27	0.231188	0.00975	0.24299	0.1962296	0.404468	0.6276
Amber03	0.20259	0.076567	0.33284	0.0115897	0.440104	0.4184
OPLSAA	0.212645	0.0764793	0.333045	0.011598	0.441724	0.492833
