# Supplementary material for: Two-dimensional monolayer salt nanostructures can spontaneously aggregate rather than dissolve in dilute aqueous solutions
Source: Nat Commun. 2021 Sep 23;12:5602. doi: 10.1038/s41467-021-25938-0 (PMC8460741; doi:10.1038/s41467-021-25938-0)
Supplement: Supplementary file 2 — Description of Additional Supplementary Files [file 41467_2021_25938_MOESM2_ESM.pdf]

Description of Additional Supplementary Files

Supplementary Movie 1. A classical molecular dynamics trajectory of the formation of the monolayer NaCl nanocrystals within monolayer water.

Supplementary Movie 2. A classical molecular dynamics trajectory of the formation of the monolayer LiCl domains within monolayer water.

Supplementary Movie 3. An *ab initio* molecular dynamics trajectory of an NaCl square nanostructure within the monolayer water confined between two graphene walls.

Supplementary Movie 4. An *ab initio* molecular dynamics trajectory of the spontaneous transformation of an NaCl domain from the hexagonal ring to a square nanostructure within the monolayer water confined between two graphene walls.

Supplementary Movie 5. An *ab initio* molecular dynamics trajectory of an LiCl hexagonal ring within the monolayer water confined between two graphene walls.

Supplementary Movie 6. An *ab initio* molecular dynamics trajectory of the spontaneous transformation of an LiCl domain from a square structure into a zigzag chain structure within the monolayer water confined between two graphene walls
